# Racial Immunity

**Published:** 1911-05-20

**Authors:** 


### Racial Immunity.

It is perhaps unfortunate that the mass of detailed facts which exist in any given department of science should cause those who are tired, as it were, of seeing only the trees, to make rather impetuous generalisations about the wood itself. Thus it comes about that a writer, such, for example, as Dr. Archdall Reid, who is laudably endeavouring to set mankind right as to the elective and selective power of alcohol and bacterial diseases, is bound to ignore a number of details which tend to undermine the security of his hypothesis. "We see, then," writes Dr. Reid, "that alcohol and opium act as agents of selection in the same way precisely as lethal and prevalent diseases. They eliminate particular types of individual. Individuals differ in their susceptibility. The more susceptible are eliminated, and as a consequence the race undergoes protective evolution."

This statement is dogmatic, and needs considerable modification. In the first place, the phrase "natural selection" is coming to be looked upon as an open sesame to every sociological problem, while in the mouths of many who use it, it is a mere shibboleth. What, after all, does the phrase mean? Simply that Nature, as opposed to an anthropomorphic Deity, selects by means of favourable variations. A favourable variation is understood to mean a variation which under the same conditions enjoys some appreciable advantage, or under changed conditions is more readily able to adapt itself to the change. This phrase, from a sociological point of view, is almost as meaningless as the much abused "survival of the fittest," which is as full of deception as the legal dictum that "a man can do what he likes with his own."

The contention that the most susceptible are eliminated, is true only to a certain extent, but there is no reason why the less susceptible should not in the second generation produce children who are as susceptible as their grandparents. Nor, again, must it be supposed that susceptibility can be dismissed with this bald statement. A man may be more susceptible at eight in the morning than at two in the afternoon. Therefore, to all intents and purposes, it is a chance whether he succumb at eight or resist at two, or whether the danger of infection at the two periods be inversely proportional. Anyone who has the slightest acquaintance with bacteriological methods must know that the apparent immunity of the fowl, for example, to anthrax is overcome by administering antipyrine. Nor is it to be supposed that those who are eliminated naturally die childless, so that there is always a chance of an inherited diathesis in the second, and supposedly less susceptible, generation.

Ample as our statistics appear to be in many cases, it is quite impossible at the present time to formulate a brilliant, all-embracing generalisation in the matter of racial immunity which shall stand for all time like the "Principia" of Newton. It seems probable that the majority of men will drink in certain given surroundings, just as under certain given conditions the young of criminals will grow up to imitate their parents and friends. The discovery of antitoxins is at least a factor of some importance in the question of protective evolution. If a permanent cure be discovered for syphilis, it ceases, save in those cases, to be an eliminative force. Even in his work, "The Laws of Heredity," Dr. Reid is trying to draw distinctions of the most futile kind. "Opium may be compared to malaria. Malaria lies midway between tuberculosis and chicken-pox."

These statements are typical of the attitude adopted by Dr. Reid in his attempt to bring everything to a common alcoholic or microbial denominator. The gradual disappearance of the Red Indian is a fact which cannot be ascribed purely to the eliminative influence of alcohol or tuberculosis. The psychological side of the question is as important. A nomadic race of hunters loses its *raison d'être* when comparative *stabilitas loci* is forced upon it.

Racial immunity as an ideal is admirable: as a fact, *pace* Dr. Reid, it is at least questionable.

